# Diagnosis of incarcerated intramesosigmoid hernia aided by multiplanar reconstruction images of multidetector computed tomography: a case report

**DOI:** 10.1186/s40792-018-0535-z

**Published:** 2018-10-20

**Authors:** Hideki Nagano, Takanori Goi, Seiichi Taguchi, Takayoshi Tsubaki, Hidemasa Uematsu

**Affiliations:** 1 Department of Surgery, Japan Community Health Care Organization Fukui Katsuyama General Hospital, 2-6-21 Nagayama-cho, Katsuyama, Fukui 911-8558 Japan; 2 First Department of Surgery, Faculty of Medicine, University of Fukui, 23-3, Matsuokashimoaizuki, Eiheiji-cho, Yoshida-gun, Fukui 910-1193 Japan; 3 Department of Radiology, Japan Community Health Care Organization Fukui Katsuyama General Hospital, 2-6-21 Nagayama-cho, Katsuyama, Fukui 911-8558 Japan

**Keywords:** Intramesosigmoid hernia, Sigmoid mesocolon hernia, MDCT, MPR

## Abstract

**Background:**

Internal hernia is a rare cause of intestinal obstruction, and sigmoid mesocolon hernia is an extremely rare form of this condition.

Among sigmoid mesocolon hernias, intramesosigmoid hernia is the least frequent subtype.

We described a case of intramesosigmoid hernia through the orifice on the right leaf of the mesosigmoid with an incarcerated ileum of 6 cm in length without strangulation. This case was diagnosed by multidetector computed tomography with multiplanar reconstruction images and treated without resection of the small intestine in a 52-year-old man with characteristic diagnostic images.

**Case presentation:**

A 52-year-old man suffering periumbilical cramping pain with sudden onset that had persisted for 1 week without recovery was referred to Fukui Katsuyama General Hospital. Multidetector computed tomography revealed small bowel obstruction, and an incarcerated short intestinal loop was revealed by sagittal slices of the multiplanar reconstruction images of the routine study of the left side of the pelvic space. Sagittal multiplanar reconstruction images also showed narrow belt-shaped fluid retention contacting the tip of the incarcerated short loop toward the cranial direction localized in the mesosigmoid. These findings indicated that the fluid and the herniated small bowel were wrapped together in the mesosigmoid, which was characteristic of intramesosigmoid hernia.

The patient underwent laparotomy operation 2 days after admission. The ileum, which was approximately 75 cm proximal to the ileocecal junction and herniated into the mesosigmoid through the right leaf, was released without resection. The orifice located in the central part of the right leaf was oval shaped and measured less than 2 cm in diameter. The left leaf of the mesosigmoid was intact. The orifice of the right lobe was closed by suture. The patient showed an uneventful recovery.

**Conclusion:**

We report an extremely rare case of incarcerated intramesosigmoid hernia that was diagnosed by multidetector computed tomography with multiplanar reconstruction images. The finding of narrow belt-shaped fluid retention contacting the tip of the incarcerated short intestinal loop is characteristic of intramesosigmoid hernia and will be useful for conclusively differentiating this disease from transmesosigmoid hernia. Although intramesosigmoid hernia is a rare cause of internal hernia, multidetector computed tomography and multiplanar reconstruction images can provide the characteristic findings and proved useful for the precise preoperative diagnosis and treatment of intramesosigmoid hernia.

## Background

Among the causes of internal abdominal hernia, sigmoid mesocolon hernia (SMH) is a very rare form of this disease and has been infrequently reported [1–7]. Although SMH is a rare disease, it is a real form of internal hernia and must be accurately diagnosed and surgically treated without delay in order to prevent strangulation, perforation, or the development of sepsis. Recently, the understanding of SMH and use of diagnostic images have expanded, thus facilitating the differential diagnosis of SMH as internal abdominal hernia.

SMH is classified into three types; among them, intramesosigmoid hernia (IMSH) is the rarest type and is characterized by a congenital, oval-shaped defect on only one leaf of the sigmoid mesocolon [8].

We describe a case of IMSH through the orifice on the right leaf of the mesosigmoid that was diagnosed by characteristic multidetector computed tomography (MDCT) and multiplanar reconstruction (MPR) images; this IMSH patient, a 52-year-old man, had an incarcerated ileum of 6 cm in size without strangulation and was treated without resection of the small intestine.

## Case presentation

A 52-year-old man with a history of appendectomy presented to his family physician suffering periumbilical cramping pain with sudden onset. He received oral medication, but the symptoms persisted.

One week later, he was referred to Fukui Katsuyama General Hospital, and his symptoms continued to persist. He received a liquid-only diet over this period without vomiting. On physical examination, he showed a pulse of 108 beats/minute, a blood pressure of 140/80 mmHg, and a body temperature of 37.2 °C. Anemia, jaundice, edema, and malnutrition were not found. Abdominal distention with mild tenderness on palpation was noted. No external hernia was present. Laboratory data showed only a mild elevation of the white blood cell (WBC) count (10.2 × 10^3^/μL) without any elevation in the C-reactive protein (CRP) level (0.12 mg/dL).

**Fig. 1:**
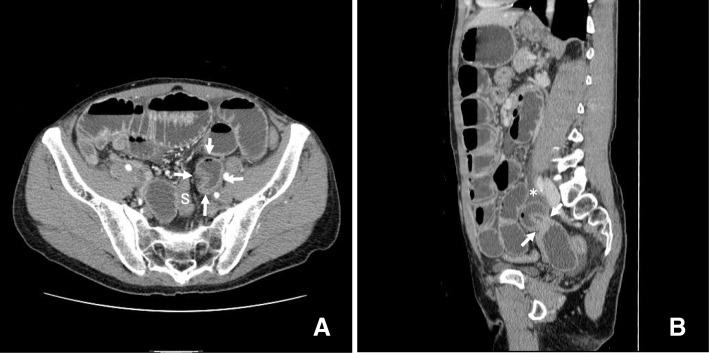
**a** MDCT showed small bowel obstruction and an incarcerated short loop of the intestine in the left pelvic space (arrows) on the left side of the collapsed sigmoid colon. S sigmoid colon. **b** Routine sagittal slices of MPR images showed two closely arranged portions of constriction (arrows) and the incarcerated section of the ileum. Narrow belt-shaped fluid retention (asterisk) was found to contact the incarcerated short loop toward the cranial direction, alongside the psoas muscle, indicating that the fluid and the incarcerated small bowel were wrapped together in the mesosigmoid, which was characteristic of IMSH

MDCT with dynamic enhanced study revealed intestinal ileus, and two closely arranged segments of constriction of the ileum were visualized by axillar slices and sagittal slices of MPR images as part of the routine study. The images showed a short closed loop sign in the left side of the pelvic space (Fig. 1a). The collapsed sigmoid colon was arranged in the ventral-dorsal direction and to the right side of the closed loop. Between the ascending and descending parts of the sigmoid colon, a right-to-left side arrangement of the intestinal mesentery was found, and the dilated intestine at the oral side of the herniated position was also observed at the right side of the sigmoid colon. The location of the incarcerated bowel suggested that the herniated orifice was located on the right side of the mesosigmoid, which was pushed to the upper-left side of the ventral-dorsally arranged sigmoid colon by the dilated intestine. The incarcerated short intestinal segment showed a good contrast effect by the enhanced study. Sagittal MPR images also showed narrow belt-shaped fluid retention on the tip of the incarcerated short loop toward the cranial direction alongside the psoas muscle, indicating that the fluid and the incarcerated small bowel were localized in the mesosigmoid (Fig. 1b). The patient was diagnosed with internal abdominal hernia, and IMSH was considered to be the most likely cause.

The patient first received non-operative treatment with peripheral infusion without oral intake. However, the obstruction did not resolve, and we decided to operate 2 days after admission.

**Fig. 2:**
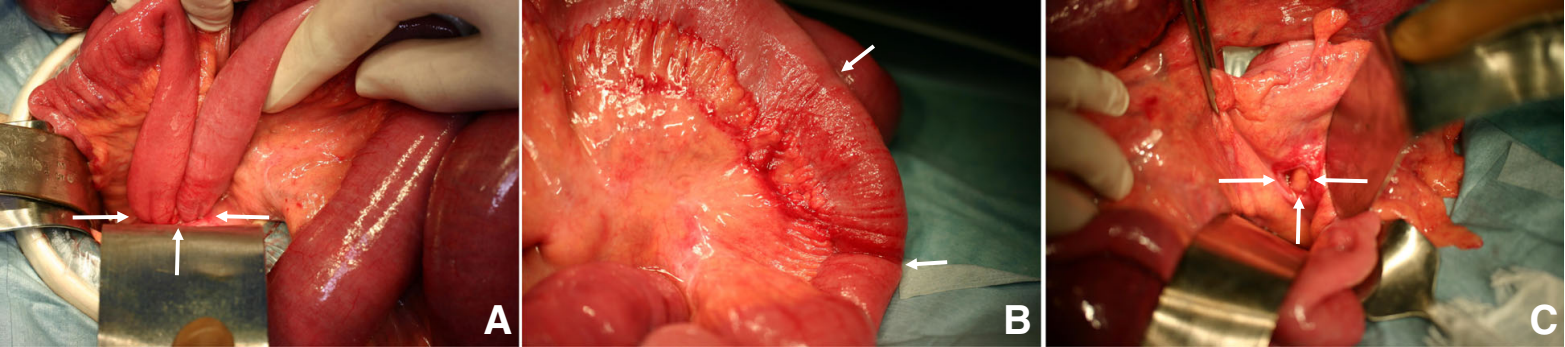
**a** Findings at laparotomy. The incarcerated small bowel was located approximately 75 cm proximal to the ileocecal junction, into the mesosigmoid through the right lobe (arrows). **b** The released ileum, measuring 6 cm in length, appeared viable and without severe color change (between the two arrows). **c** The incarcerated ileum was gently released. The orifice located at the central part of the right lobe of the mesosigmoid was oval shaped and measured less than 2 cm in diameter (arrows). The intact inside of the left lobe was seen through the orifice

A long tube was inserted, and the patient underwent laparotomy operation. Dilated intestine and serous ascites were observed; the ileum was approximately 75 cm proximal to the ileocecal junction and was found to be herniated into the mesosigmoid through the right leaf (Fig. 2a). The opposite side of the mesosigmoid was bulged by the incarcerated ileum and wrapped in the left leaf, without exposure of the ileum through the mesocolon. Attachment of the lateral aspect of the sigmoid mesocolon to the parietal peritoneum was normal. The incarcerated ileum was gently released by a pressing maneuver from the left leaf. The released ileum, 6 cm in length, appeared viable and without color change, and thus, resection of part of the ileum was not conducted (Fig. 2b). The orifice located at the central part of the right leaf was oval shaped and measured less than 2 cm in diameter (Fig. 2c). The left leaf of the mesosigmoid was intact. The orifice of the right leaf was closed by suture. After an uneventful recovery, the patient was discharged from the hospital 12 days after the operation.

**Fig. 3:**
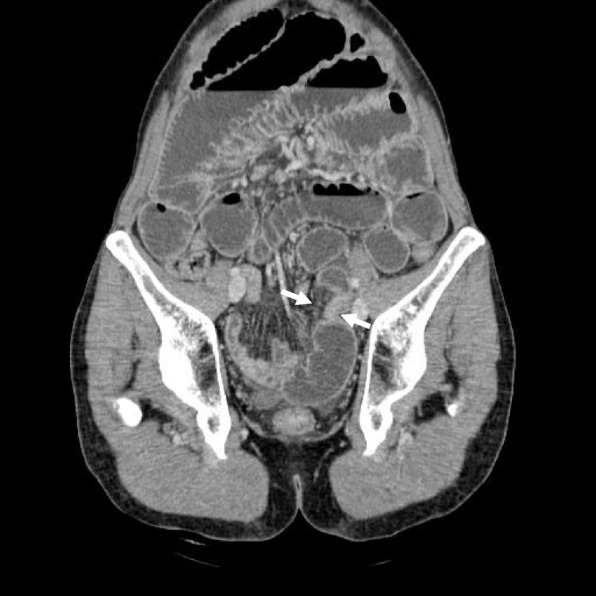
Reconstructed oblique coronal image of MDCT showed the constricted part of the intestine and the converged mesentery (arrows), reflecting the position of the hernia orifice

We investigated the preoperative MDCT and reconstructed oblique coronal images using the SYNAPSE VINCENT® workstation (Fujifilm Medical Co., Ltd., Tokyo, Japan) after surgery. Oblique coronal images revealed the incarcerated intestine and the converging mesenteric vessels to indicate the hernia orifice more clearly, which measured 18 mm in width (Fig. 3).

### Discussion

Internal hernia represented by paraduodenal hernia, pericecal hernia, and others is a rare cause of intestinal obstruction and is estimated as responsible for only 0.5–0.9% of intestinal obstruction in patients with no history of abdominal surgery [9, 10]. Among internal hernia cases, SMH, herniation involving the sigmoid mesocolon, is a rare subset and is estimated at approximately 6% of all internal hernia cases [11]. Benson and Killen classified SMHs into three types: intersigmoid hernia (ISH), transmesosigmoid hernia (TMSH), and IMSH [8]. ISH has been defined as herniation into the peritoneum fossa due to insufficient attachment of the left lobe of the mesosigmoid to the parietal peritoneum of the posterior abdominal wall, and TMSH and IMSH are herniation into abnormal (mostly congenital) oval-shaped defects of one side or both lobes of the mesocolon. IMSH is described as the rarest type of SMH (ISH 30/34, TMSH 3/34, IMSH 1/34, respectively) [8].

Of the three types of SMH, TMSH showed the highest rate of bowel resection (50%) compared with ISH (18.8%) and IMSH (13.9%) [3]. SMH should be accurately diagnosed without delay, considering the risk of strangulation.

Regarding the modality to facilitate a precise diagnosis, MDCT is most useful; ISH cases should be visualized such that the small bowel goes across the ventral part of the sigmoid colon once, and then, the incarcerated small loop is formed from the lower-left abdomen to the cranial side because the hernia orifice of ISH is located at the attachment of the lateral aspect of the sigmoid mesocolon. In cases of TMSH and IMSH, as for the congenital oval-shaped foramen of the sigmoid mesocolon that can occur in both lobe sides, it is necessary to consider that the short bowel could herniate into or through the mesosigmoid from either side. However, it is difficult to preoperatively distinguish between IMSH and TMSH by MDCT.

In the presented case, routine sagittal MPR imaging showed narrow belt-shaped fluid retention contacting the tip of the herniated short loop of the intestine. This indicated that the fluid and the herniated small bowel were wrapped together in the mesosigmoid, which precisely indicated IMSH. This finding seems to be characteristic of IMSH and may be useful for conclusively differentiating this disease from TMSH.

Our case showed the characteristic findings noted above by routine sagittal MPR images and provided sufficient information on the IMSH before the operation; however, it is difficult to obtain high-quality image views of diagnostic value by routine images alone in many cases. Kumagai [12] reported the usefulness of oblique multiplanar images on MDCT, and we investigated the preoperative MDCT and reconstructed oblique coronal images using the SYNAPSE VINCENT® workstation after surgery to visualize the hernia orifice and illustrate a more detailed anatomical placement. Oblique coronal images revealed the incarcerated intestine and the converging mesenteric vessels that more clearly indicate the hernia orifice in the mesosigmoid. Preoperative investigation of oblique multiplanar images on MDCT can help physicians understand the three-dimensional placement of the bowels of interest to facilitate accurate diagnosis of SMH.

Shibuya [13] reported the usefulness of a laparoscopic operation for ISH. Regarding IMSH cases, application of the laparoscopic approach has not been rigorously reported. It seems that further study of the laparoscopic operation for IMSH is necessary, and the precise preoperative understanding of the anatomical placement of the bowel of interest would contribute to its safe application.

There are two points of reflection on the treatment course of this case. First, despite being able to diagnose IMSH from the images, surgery could not be performed immediately. Due to the rarity of this disease and the inexperience with image findings, we could not share our understanding of the disease condition among surgeons, and therefore, we were not able to comply with the emergency surgery policy. This report was motivated by our reflection and the need to publish on this disease and describe its characteristic imaging findings. A long tube was inserted before the operation using a routine technique at the time of an intestinal obstruction operation at our facility; however, it is thought that this will not be routinely necessary in the future because there are many reports in which a long tube was not used for IMSH operations, and the clinical course thereafter did not show any problems.

## Conclusions

We reported an extremely rare case of incarcerated IMSH that was diagnosed by MDCT with MPR images. The finding of narrow belt-shaped fluid retention contacting the tip of the incarcerated short intestinal loop is characteristic of IMSH and will be useful for conclusively differentiating this disease from TMSH. Although IMSH is a rare cause of internal hernia, MDCT and MPR images can provide the characteristic findings and are useful to precisely diagnose and treat IMSH.

## Abbreviations

CRP: C-reactive protein
IMSH: Intramesosigmoid hernia
ISH: Intersigmoid hernia
MDCT: Multidetector computed tomography
MPR: Multiplanar reconstruction image
SMH: Sigmoid mesocolon hernia
TMSH: Transmesosigmoid hernia
WBC: White blood cell
